# Discharge of an Ovum and Its Relation in Point of Time to Menstruation

**Published:** 1875-11

**Authors:** 


The Discharge of an Ovum, and its Relation in Point of Time to Menstruation.—It is still a vexed question among physiologists at what period the human ovary discharges its products into the Fallopian tubes. The general belief is that the ova are thrown off towards the end of the menstrual flow or immediately after its cessation. Dr. John Williams, Assistant Obstetric Physician to University College Hospital, however, in a note communicated to the Royal Society, and published in Proceedings No. 162, brings forward a number of cases which tend to prove that the discharge of ova really occurs before the appearance of the monthly flow, with which it is connected. In ten out of fourteen bodies of women dying of various diseases, either a day or two before, or two or three weeks after, the ordinary catamenial period, rupture of a Graafian follicle, or hemorrhage into its cavity, had occurred before the return of the menses. Of the other cases—in one it was doubtful whether rupture of a follicle or the appearance of the discharge would have taken place first; in two a menstrual period had passed without maturation of a follicle; and in the fourth a periodical discharge was imminent, though the ovaries contained no matured Graafian follicle. Dr. Williams finds that other writers, who have recorded cases similar to his, in the main favor the view he supports. Reicher, however, who has examined the bodies of twenty-three women, in whose organs signs of mentruation were recognizable, arrived at the conclusion that rupture of the Graafian follicle takes places at an early stage of the menstrual flow.—*Medical Times and Gazette—The American Journal of the Medical Sciences.*

